# Probing Structure and Function of Alkali Sensor IRR with Monoclonal Antibodies

**DOI:** 10.3390/biom10071060

**Published:** 2020-07-16

**Authors:** Alexander S. Goryashchenko, Andrey A. Mozhaev, Oxana V. Serova, Tatiana N. Erokhina, Alexander N. Orsa, Igor E. Deyev, Alexander G. Petrenko

**Affiliations:** 1 Laboratory of Receptor Cell Biology, Department of Peptide and Protein Technologies, Shemyakin and Ovchinnikov Institute of Bioorganic Chemistry of Russian Academy of Sciences, 117997 Moscow, Russia; a.a.mozhaev@gmail.com (A.A.M.); oxana.serova@gmail.com (O.V.S.); saniaorsa@gmail.com (A.N.O.); petrenkoag@gmail.com (A.G.P.); 2 Laboratory of Bioorganic Structures, Department of X-ray and Synchrotron Studies, A.V. Shubnikov Institute of Crystallography of Federal Scientific Research Centre “Crystallography and Photonics” of Russian Academy of Sciences, 119333 Moscow, Russia; 3 Laboratory of Molecular Diagnostics, Department of Plant Molecular Biology and Biotechnology, Shemyakin and Ovchinnikov Institute of Bioorganic Chemistry of Russian Academy of Sciences, 117997 Moscow, Russia; erokhina@yahoo.com; 4 Group of Molecular Physiology, Department of Peptide and Protein Technologies, Shemyakin and Ovchinnikov Institute of Bioorganic Chemistry of Russian Academy of Sciences, 117997 Moscow, Russia; deyevie@gmail.com

**Keywords:** IRR, monoclonal antibody, activator, inhibitor, receptor tyrosine kinase, alkali sensor

## Abstract

To study the structure and function of the pH-regulated receptor tyrosine kinase insulin receptor-related receptor (IRR), а member of the insulin receptor family, we obtained six mouse monoclonal antibodies against the recombinant IRR ectodomain. These antibodies were characterized in experiments with exogenously expressed full-length IRR by Western blotting, immunoprecipitation, and immunocytochemistry analyses. Utilizing a previously obtained set of IRR/IR chimeras with swapped small structural domains and point amino acid substitutions, we mapped the binding sites of the obtained antibodies in IRR. Five of them showed specific binding to different IRR domains in the extracellular region, while one failed to react with the full-length receptor. Unexpectedly, we found that 4D5 antibody can activate IRR at neutral pH, and 4C2 antibody can inhibit activation of IRR by alkali. Our study is the first description of the instruments of protein nature that can regulate activity of the orphan receptor IRR and confirms that alkali-induced activation is an intrinsic property of this receptor tyrosine kinase.

## 1. Introduction

Receptor tyrosine kinases are one of the key components that provide intercellular interaction and response to various extracellular signals. These receptors are transmembrane proteins that bind polypeptide ligands and play an important role in cell growth, differentiation, and metabolism. Dysfunction of these receptors can lead to emergence and development of cancer [1]. Typically, receptor tyrosine kinases consist of three domains—extracellular hydrophilic domain, which provides recognition of the ligand, hydrophobic transmembrane domain, which provides integration of the receptor in the lipid bilayer of the cell membrane, and the cytoplasmic catalytic domain, which transmits the signal inside the cell [2]. Most of receptor tyrosine kinases are monomeric molecules that undergo oligomerization (primarily dimerization) as a result of interaction with the ligand, which leads to convergence and autophosphorylation of tyrosine residues in the intracellular part of the receptor, as well as to conformational changes that stabilize the activated form of the receptor [3]. Phosphotyrosine residues then serve to recruit intracellular signaling proteins, mainly through Src homology-2 (SH2) or phosphotyrosine-binding (PTB) domains [4].

The exception is three members of the insulin receptor family, which in the inactive state are already dimers connected by disulfide bridges and, upon activation, change their conformation. This family includes three highly homologous receptors: the insulin receptor (IR), the insulin-like growth factor receptor (IGF-IR), and the insulin receptor-related receptor (IRR). These receptors show the same domain structure. Their monomers are split by proteolysis in two subunits, α and β, that form a covalent complex [5]. Thus, the mature receptors consist of four polypeptide chains. The identical α-subunits consist of two leucine-rich L-domains separated by a cysteine-rich domain, followed by the fibronectin type III (FnIII-1) domain and part of another FnIII domain (FnIII-2). The β-subunits contain the second part of the FnIII-2 domain and the third FnIII domain (FnIII-3), followed by the transmembrane region. Their tyrosine kinase domain is located in the cytoplasmic part of the receptor β-subunit [6].

Whereas IR and IGF-IR structure and functions are thoroughly studied, little is known about their close homolog IRR. IRR was originally discovered in DNA cloning experiments due to its homology to IR [7]. No peptide or protein agonist of IRR have been found since then that significantly complicated its functional analysis. Recent in vitro studies together with anatomical characterization and knockout animal experiments led to the conclusion that IRR is an extracellular pH sensor with a role in the regulation of the acid-base balance by the kidneys [8,9].

The analysis of IRR transfected cells revealed receptor activation by mildly alkaline extracellular media. Two other members of the IR family did not show such a response [8]. Activation of IRR by alkaline media caused specific and dose-dependent conformational changes in the ectodomain of the receptor, leading to autophosphorylation of intracellular kinase domains [10]. As a result of IRR activation, phosphorylation of intracellular signal protein IRS-1 (Insulin receptor substrate 1) and protein kinase B (Akt/PKB) was observed [11,12]. In vivo experiments in knockout mice revealed the role of IRR in the regulation of excess bicarbonate excretion by the kidneys under experimentally induced alkalosis [8,12]. These mice also showed behavioral abnormalities [13,14].

Since IRR activation presumably does not involve interaction with a substance of a significant size, it represents an interesting object for structural and functional studies. We now report that, with the recombinant extracellular region of human IRR as an antigen, we prepared and characterized a panel of monoclonal antibodies. We determined the antibody binding domains within exogenously expressed full-length IRR utilizing a previously produced set of IRR point mutants and its chimeras with IR (Figure S1) [15,16]. We found that 4D5 antibody that binds to the Fn(III)-1 domain of IRR can work as its agonist at neutral pH whereas the application of Fn(III)-2/3-binding 4C2 antibody results in the inhibition of alkali-induced IRR activation. These findings confirm the role of IRR as a pH sensing membrane protein and point to location of the alkali-sensing machinery within multiple domains of its extracellular region.

## 2. Materials and Methods

### 2.1. Monoclonal Antibodies (mAbs) Production and Purification

For mouse immunization, 100 µg ectoIRR [17,18], mixed with an equal volume of Freund’s complete adjuvant (MP Biomedicals, Irvine, CA, USA), was injected intraperitoneally and boosted twice with the same dose mixed with incomplete adjuvant at 2 weeks intervals. The final injection of 100 µg antigen was without adjuvant. Immunized spleen cells were fused 3 days later with the mouse myeloma cell line Sp2/0 by using 45% polyethylene glycol (Sigma, St. Louis, MO, USA). Cells were cultured under selective conditions on Dulbecco’s HAT MEM (Gibco, Waltham, MA, USA) supplemented with 15% fetal calf serum (Gibco, Waltham, MA, USA) in the presence of mouse peritoneal macrophages as feeder cells [19].

Culture fluids were screened for specific antibody production by indirect ELISA with 5 µg/mL ectoIRR as antigen. Hybridomas secreting specific mAbs were cloned twice under limiting dilution conditions. mAbs were purified from ascitic fluids by affinity chromatography on protein G Sepharose 4 Fast Flow (Amersham Biosciences, Little Chalfont, Buckinghamshire, UK).

### 2.2. Immunoprecipitation

HEK293T cells were grown on a Dulbecco’s modified Eagle’s medium (DMEM) containing 10% FBS, 1% penicillin/streptomycin, and 2 mM L-glutamine under standard conditions (37 °C and 5% CO_2_). Before transfection, cells were seated on a 24-well plate covered with poly-L-Lysine. Cells were transfected with plasmids based on the vector pcDNA 3.1, encoding the mentioned above mutant forms of IRR, as well as wild type human IRR, IR and IGF-IR, and mouse IRR, using the Transfectin-56 reagent (Unifect Group, Moscow, Russia) according to the manufacturer’s instructions. After transfection, the cells were grown for two days, washed with PBS, and lysed using 700 µL of cooled to 4 °C buffer consisting of 20 mM Tris-HCl (pH 8.0), 150 mM NaCl, 1.5% Triton X-100, 2 mM EDTA, 1 mM PMSF. The lysates were then centrifuged at 15,000 RPM and 4 °C for 30 min and the supernatants were taken into new tubes, leaving 80 µL of aliquots as positive control. Then, 3 µg of antibodies against the extracellular part of IRR (1D2, 4D5, 4C2, 4A2, 3C5, 3B4) were added to the supernatants and incubated at 4 °C for 2 h. Then, 50 µL of Protein G-sepharose (Bialexa, Moscow, Russia) were washed twice with PBS and three times with lysis buffer, then added to a mixture of supernatant and antibodies, and incubated at 4 °C overnight. On the next day, sepharose was centrifuged, a supernatant was removed. Then protein G-sepharose was washed three times with lysis buffer and 100 µL of SDS-Loading Buffer for electrophoresis was added to the precipitate, samples were heated to 95 °C for 10 min, after which the supernatant was taken and used for electrophoresis followed by Western blotting analysis.

### 2.3. SDS-PAGE and Western Blotting

HEK293T cells were grown on a DMEM medium containing 10% FBS, 1% penicillin/streptomycin, and 2 mM L-glutamine under standard conditions (37 °C and 5% CO_2_). Before transfection, the cells were seeded on a 24-well plate covered with poly-L-Lysine. Cells were transfected with plasmids based on the vector pcDNA 3.1, encoding the mentioned above mutant forms of IRR, as well as wild type human IRR, IR and IGF-IR, and mouse IRR, using the Transfectin-56 reagent (Unifect Group, Moscow, Russia) according to the manufacturer’s instructions. After transfection, the cells were grown for two days, washed with PBS, and lysed with 200 µL of the 1.5× SDS-Loading Buffer for electrophoresis. Cell lysates, eluates from the immunoprecipitation, and purified ectoIRR protein were separated by SDS-PAGE in 8% gel followed by blotting onto ECL-grade nitrocellulose (Amersham, Little Chalfont, Buckinghamshire, UK). Nonspecific adsorption of proteins was prevented by incubating the membranes in Tris-buffered saline with Tween (TBST) buffer (50 mM Tris-HCl, pH 8.0, 150 mM NaCl, 0.05% Tween-20) containing 5% non-fat milk for 1 h. The blots were further incubated with primary antibodies overnight. To identify IRR, we used primary rabbit polyclonal antibody anti-C-end-IRR against the C-terminal cytoplasmic domain of IRR (961-1297 a.a.) that can detect both phosphorylated and non-phosphorylated forms of the receptor [8,15]. Then, primary antibodies were washed off, followed by the addition of secondary donkey antibodies against rabbit IgG conjugated with horseradish peroxidase (Jackson ImmunoResearch, Cambridge House, St. Thomas’ Place, Cambridgeshire, UK). The bands were visualized by means of luminescent substrate SuperSignal West Pico (Thermo Fisher Scientific, Waltham, MA, USA) on Fusion Solo device and quantitative analysis was performed using Fusion software (Vilber Lourmat, Collégien, France).

### 2.4. Immunocytochemistry

HEK293T cells were grown on a DMEM medium containing 10% FBS, 1% penicillin/streptomycin, and 2 mM L-glutamine under standard conditions (37 °C and 5% CO_2_). Before transfection, cells were seated on a 24-well plate covered with poly-L-Lysine. Cells were transfected with plasmids based on the vector pcDNA 3.1, encoding the mentioned above mutant forms of IRR, using the Transfectin-56 reagent (Unifect Group, Moscow, Russia) according to the manufacturer’s instructions. After transfection, the cells were grown for two days, then they were washed with PBS and fixed with a mixture of methanol/acetone 1:1 *v*/*v* for 20 min at -20 °C. Non-specific antibody adsorption was prevented by incubating the cells in PBS containing 2% BSA for 3 h at room temperature. The cells were then incubated with 3 µg of primary antibody solutions against the extracellular part of IRR (1D2, 4D5, 4C2, 4A2, 3C5, 3B4) in PBS per well at 4 °C overnight. The washing of unbound antibodies was performed by PBS three times for 7 min, after which secondary goat antibodies against mouse IgG were added, conjugated with Cy3 (Jackson ImmunoResearch, Cambridge House, St. Thomas’ Place, Cambridgeshire, UK), dissolved in PBS at a ratio of 1:5000. Incubation with secondary antibodies was carried out for 1 h at room temperature, after which the cells were washed from unbound antibodies with PBS three times for 7 min. The fluorescence of the cells was detected using Olympus IX51 microscope (Olympus, Shinjuku, Tokyo, Japan) equipped with a 60× oil-immersion objective and a set of filters for Cy3 fluorescence.

### 2.5. Effect of Monoclonal Antibodies on IRR Activation

The following experiment was conducted to study the effect of monoclonal antibodies on the activation of IRR by a slightly alkaline medium. After transfection of HEK293 cells with a plasmid cDNA encoding human IRR, the DMEM medium was replaced by serum free medium F-12 containing monoclonal antibodies 4D5, 4С2, 3С5, and 1D2. After incubation of cells with antibodies for 1 h, the medium was replaced with 40 mM Tris-HCl solution, pH 7.4 or 9.0, following by the incubation for 15 min. After that, the medium was removed and SDS electrophoresis sample buffer was added immediately. Then, total cell lysates were analyzed by Western blotting using polyclonal rabbit antibodies (anti-pIRR) obtained earlier in our laboratory, specifically staining the phosphorylated tyrosine kinase IRR domain, as well as polyclonal rabbit anti-C-end-IRR specific to the C-terminal cytoplasmic domain (961–1297 a.a.) that can detect both phosphorylated and non-phosphorylated forms of the receptor [8,15]. To check the downstream pathways activation, HEK293 cells were transfected with the plasmid cDNA encoding human IRR, the DMEM medium was replaced by serum free medium F-12 containing 90 µg/mL of monoclonal antibodies 4D5 and 4С2. After incubation of cells with antibodies for 10 min, 1 M Tris-HCl buffer with pH 7.4 or 9.0 was added to the medium to the final concentration of 50 mM, followed by the incubation for 15 min. After that, the medium was removed and SDS electrophoresis loading buffer was added immediately. Then, total cell lysates were analyzed by Western blotting using rabbit polyclonal antibodies against the C-terminus of IRR and pIRR obtained in our laboratory [8,15], anti-IRS-1 (Upstate Biotechnology, Lake Placid, NY, USA), anti-pIRS-1(Y612) (Thermo Fisher Scientific, Waltham, MA, USA), anti-ERK1/2 (Cell Signaling Technology, Danvers, MA, USA), and anti-pERK (Cell Signaling Technology, Danvers, MA, USA). The bound antibodies were detected by treatment with a chemiluminescent substrate Super Signal West Pico (Thermo Fisher Scientific, Waltham, MA, USA) and visualized with Fusion Solo system (Vilber Lourmat, Collégien, France).

## 3. Results

### 3.1. Purification of IRR Ectodomain and Preparation of Monoclonal Antibodies

Human IRR ectodomain was produced and purified as described in [17,18]. Briefly, CHO-K1 cells were transfected with the ectoIRR encoding plasmid and stable expression clone of CHO-K1 was obtained. The cells were grown in serum free media that was collected and ectoIRR was purified by anion-exchange, gel filtration, and hydrophobic chromatography.

Monoclonal antibodies were obtained by injecting mice with purified IRR ectodomain and further clonal selection according to the standard hybridoma technology as described in Section 2. mAbs were selected based on their strong positive reaction with the antigen in indirect ELISA and on immunoblots. As a result of two fusion experiments, six hybridoma cell lines: 1D2, 3B4, 3C5, 4A2, 4C2, and 4D5, producing specific mAbs to ectoIRR protein were generated.

### 3.2. Western Blotting

We tested the ability of the generated antibodies to work in Western blotting with human IRR (hIRR)- and mouse IRR (mIRR)-expressing HEK293T cells and with the purified hIRR ectodomain [18,20] under reducing and non-reducing conditions. Under reducing conditions only, the 4D5 antibody stained IRR and its ectodomain (Figure S2). Without a reducing agent, none of the antibodies stained the full-length IRR, whereas 1D2, 4C2, and 3B4 only decorated the ectodomain (Figure S3). The results are summarized in Table 1. It can be concluded that 1D2, 4C2, and 3B4 antibodies have conformational epitopes which disappear under reducing conditions. On the contrary, 4D5 antibody has a linear epitope, but it seems unusual that it cannot stain ectoIRR without a reducing agent, despite having been raised using non-reduced protein. A possible explanation is that, upon reduction, a small fragment of the IRR beta subunit gets separated from the alpha subunit due to cystine reduction, and this process unmasks the 4D5 epitope. In immunized animals, antigens typically undergo multiple cleavages in lysosomes and fragments without cysteines/cystines have higher antigenicity. None of the antibodies stained murine IRR. In these experiments, polyclonal anti-IRR ectodomain antibody [15] was used as a positive control.

The 3B4 antibody showed no binding to the IRR in all immunoprecipitation and immunocytochemistry experiments, whereas in ELISA (see Figure S4) and Western blotting (see Table 1 and Figures S2 and S3) it recognizes ectoIRR, but not the reduced ectoIRR form.

### 3.3. Immunoprecipitation

The obtained monoclonal antibodies were tested using human IRR with substitution of its extracellular domains to similar ones of IR or with point mutations (Figure S1) as well as wild type human IRR, IR, IGF-IR, mouse IRR, and purified soluble human IRR ectodomain (ectoIRR) [20]. We presumed that mutants with the substitution of the whole structural domains can help us to locate the binding sites of the antibodies at the domain scale, while the mutants with point substitutions may give us information about the position of binding sites inside the IRR domains. Experiments were carried out to identify the binding centers of six monoclonal antibodies against IRR. For this purpose, HEK293T cells were transfected with plasmids encoding human IR, human IGF-IR, and mouse IRR as well as wild-type full-size human IRR and its 10 mutant forms, which contained point mutations or substitution of selected domains with their analogs of the insulin receptor. The obtained cells were lysed and the antibody binding to wild-type and mutant forms of IRR and also its cross-reactivity with human IR, human IGF-IR, and mouse IRR was tested by immunoprecipitation with protein G-sepharose. Detection of bound receptor forms was performed by Western blotting with the polyclonal antibody against the C-terminal cytoplasmic domain of IRR (961–1297 a.a.) that can detect both phosphorylated and non-phosphorylated forms of the receptor [8,15]. The obtained results (see Figure S5) are summarized in Table 2, marked with red symbols. These results demonstrate that 1D2 antibody binds to the L1C-domain, 3C5 to the one of fibronectin domains, 4D5 to the Fn(III)-1 domain, and 4C2 and 4A2 bind to the Fn(III)-2/3 domains. Binding of the 3B4 antibody to any of the tested constructs was not observed. In addition, it was shown that there is no cross-reactivity of the monoclonal antibodies against human IRR with human IR and IGF-IR and with mouse IRR (see Figures S7–S9).

### 3.4. Immunocytochemistry

Similar complementary tests were performed utilizing immunofluorescence of transfected cells. Although receptor proteins were intact, as in the immunoprecipitation experiments, they remained in their native membrane environment. The results obtained are shown in Table 2 and Figure S6, and marked with blue symbols.

As in the case of immunoprecipitation, it was shown that there is no cross-reactivity of the monoclonal antibodies against human IRR with human IR and IGF-IR and with mouse IRR (Data not shown).

The data obtained by two different methods allowed us to conclude that 1D2 antibody binds to the L1C-domain, 3C5 to the Fn(III)-1 domain. 4D5 antibody binds to the first fibronectin domain, while 4C2 and 4A2 bind to the second and third fibronectin domains. 3B4 bound to the original antigen but did not show any detectable binding to all constructs tested suggesting that its epitope exposed in IRR ectodomain is masked when the ectodomain is attached to the membranous part of IRR. Additionally, the Western blotting results indicate that the 3B4 epitope in the soluble IRR ectodomain is sensitive to SDS denaturing in reducing conditions.

In several cases, the results of immunoprecipitation were positive, while immunocytochemistry showed no binding. 4C2 and 4A2 bind to the second and third fibronectin domains according to immunoprecipitation data. Yet, 4C2 does not bind to Fn(III)-1 mutant whereas 4A2 does not bind to Fn(III)-1, L2, MVD, PV, and RL-Fn mutants in immunocytochemistry experiments.

**Figure 1:**
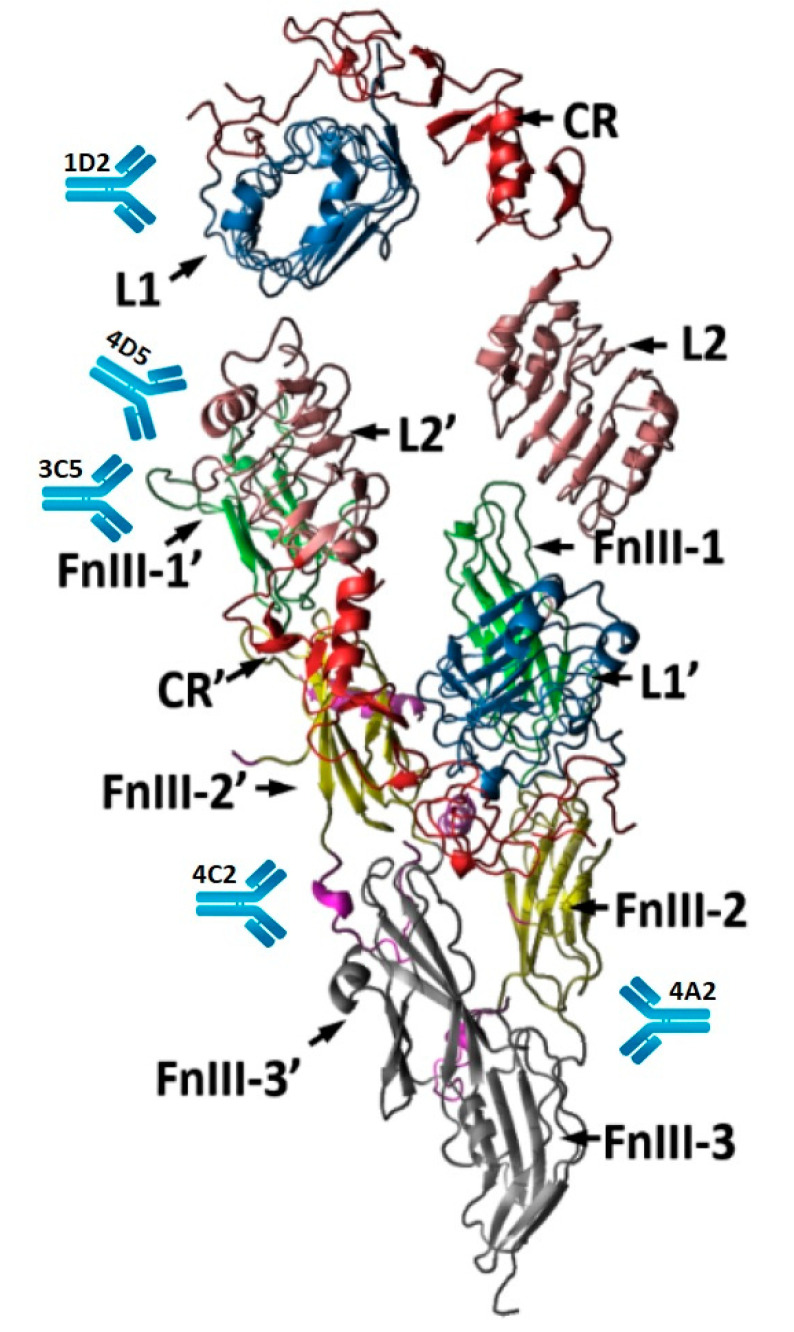
Scheme of the binding of monoclonal antibodies to IRR. Structure was taken from [18].

Thus, it is possible to propose the following diagram of the binding of monoclonal antibodies to the recently reported drop-like form of IRR (Figure 1).

### 3.5. Influence of the Monoclonal Antibodies on the IRR Activation by Mildly Alkaline pH

**Figure 2:**
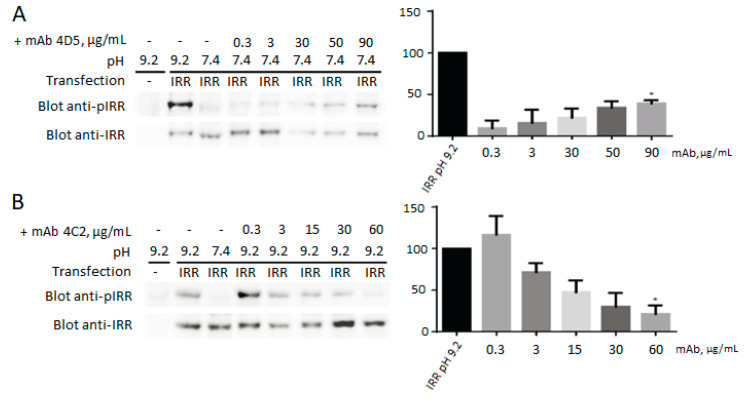
Activation and inhibition of IRR by monoclonal antibodies. For each antibody the IRR phosphorylation level at pH 9.2 is indicated as 100%. Transfected cells were incubated with different concentrations of antibodies from 0.3 to 90 µg/mL. Lysates of transfected cells were directly analyzed by Western blotting with anti-pIRR antibodies and after stripping with anti-IRR antibodies. For the quantitative analysis of Western blots, we used Fusion Solo system (Vilber Lourmat, Collégien, France). The ratio of integral density of the phosphorylated receptor (pIRR signal) to the total receptor (IRR antibody signal) was plotted versus antibody concentration. Values are means ± SE (*n* ≥ 4). Asterisks indicate *p* < 0.05. (**A**) Incubation of the IRR-expressing HEK293 cells with 4D5 antibodies at рН 7.4 leads to IRR phosphorylation. (**B**) Incubation of the IRR-expressing HEK293 cells with 4C2 antibodies at рН 9.2 leads to the inhibition of the IRR activation by alkali.

It was shown that 3С5, 4A2, 3B4, and 1D2 antibodies have no effect on IRR activation, but, in media with neutral pH, 4D5 antibody is capable of activating IRR, and incubation with antibody 4C2 leads to inhibition of the IRR activation in slightly alkaline medium (Figure 2).

**Figure 3:**
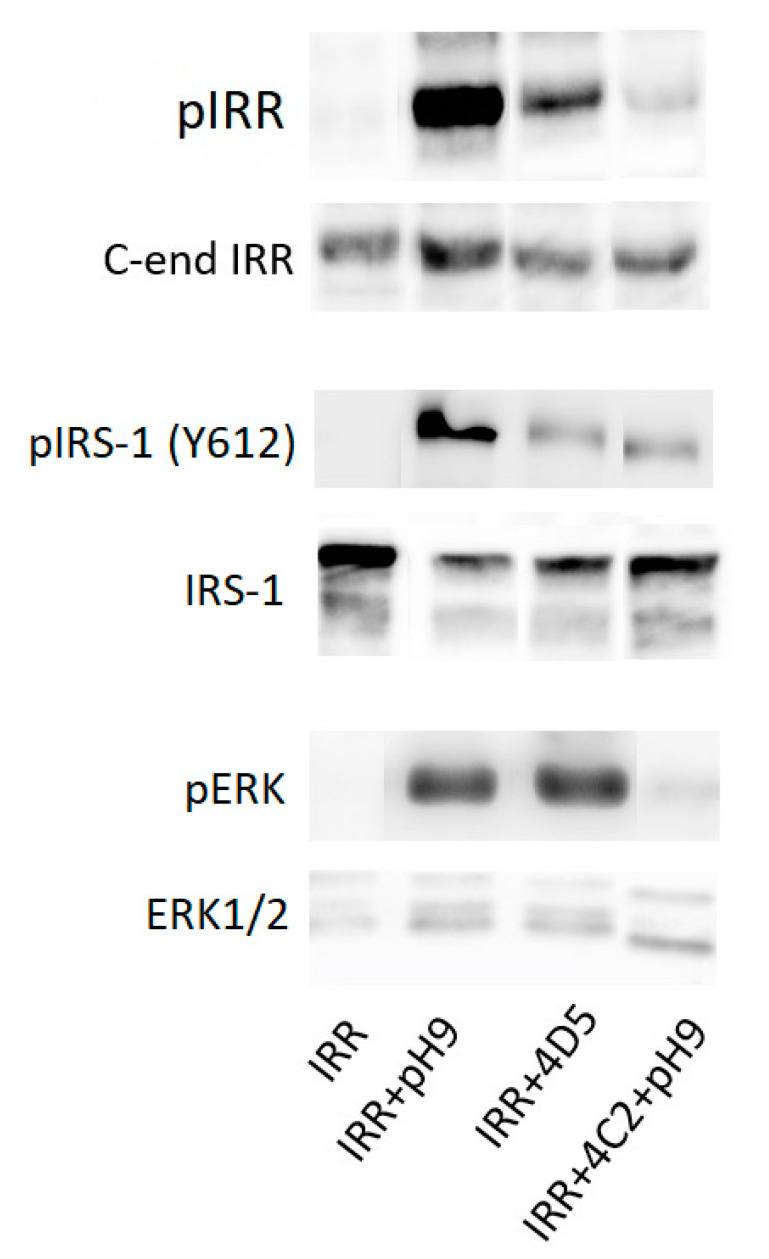
Activation and inhibition of the IRR downstream signaling pathways in IRR-transfected HEK293 cells.

Moreover, 4D5 antibody activates not only the IRR itself, but two downstream signaling adapters—IRS-1 and ERK1/2—while 4C2 antibody treatment in alkaline media results in the inhibition of both these pathways (Figure 3). Notably, it was previously shown that ERK1/2 is not activated during the alkaline treatment of IRR-transfected CHO cells [8], whereas here we demonstrate that in HEK293 cells ERK activation occurs both in the case of alkali and 4D5 antibody treatment.

**Figure 4:**
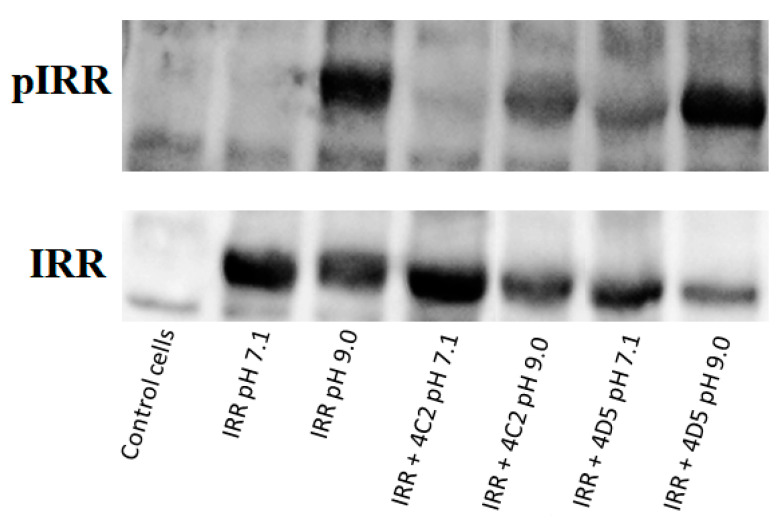
Influence of 4D5 and 4C2 antibodies (90 µg/mL) on IRR activation at neutral and alkaline pH.

We also studied the influence of 4D5 and 4C2 antibodies on IRR activation at pH 7.4 and 9.0. Therefore, incubation of the IRR-expressing HEK293 cells with 4C2 antibody at neutral pH has no effect, but if such incubation is followed by the change of pH to 9.0, 4C2 partially inhibits IRR activation (Figure 4). In the case of the 4D5 antibody, as it was stated before, incubation of the IRR-expressing HEK293 cells with this mAb at neutral pH leads to the partial activation of IRR. If such incubation is followed by the change of pH to 9.0, the level of phosphorylated IRR is significantly higher than in case of separate treatment of cells with alkaline pH or 4D5 antibody (Figure 4). This result indicates synergism of alkaline media and 4D5 antibody effects on IRR activation.

Thus, 4D5 antibody acts as an IRR agonist, and 4C2 as a reverse agonist. These effects of 4C2 and 4D5 antibodies can then be applied in the structure-function studies.

Further immunoprecipitation experiments demonstrated that preliminary incubation of IRR-expressing cells in F-12 medium with pH 8.9 does not affect the binding of antibodies (see Figure S10), therefore IRR phosphorylation does not change the structure of the mAbs epitopes.

## 4. Discussion

Receptor tyrosine kinase IRR is a close relative of the insulin receptor. They have identical domain structures and high protein sequence homology, but they can be activated by totally different ligands, the hydroxyl-anion and the insulin, respectively, without any cross-reactivity. We have previously demonstrated that IRR activation is defined by its extracellular region, involves multiple domains, and shows positive cooperativity with two synergistic sites. In this report we obtained additional tools for investigation of IRR pH sensing phenomena. In this study, we characterized six mouse monoclonal antibodies against the IRR ectodomain by Western blotting, immunoprecipitation, and immunocytochemistry. It was shown that 1D2 antibody binds to the L1C domain, 3C5 and 4D5 to the Fn(III)-1 domain, 4A2 and 4C2 to the Fn(III)-2/3 fibronectin domains of IRR. In addition, we showed that none of these antibodies could bind to mouse IRR, human IR, and human IGF-IR. It should also be noted that 3B4 antibody binds only to the purified IRR ectodomain in Western blotting but not to cell-expressed full-size IRR. Taken together, our data indicate that all mAbs have different patterns of interaction with IRR, indicating specific epitopes for each of them.

It is known that monoclonal antibodies can be used as activators or inhibitors of receptor tyrosine kinases [21]. Several monoclonal antibodies against IR and IGF-IR were described. Thorough work in this field was done by the Siddle lab by producing and characterizing a number of mAbs that can activate or inhibit IR and IGF-IR [22,23,24]. Moreover, epitopes of several antibodies were defined [23,25].

Recent studies reported a few more anti-IR mAbs focused on their potential therapeutic application. The antibody named XMetA is the IR activator. Another antibody XMetS does not directly activate IR but enhances the receptor’s affinity to the insulin, and, finally, XMetD is an inhibitor of IR signaling [26]. As for the IGF-IR, there are several human or humanized anti-IGF-IR monoclonals. Several reports showed that these antibodies inhibit IGF-IR activity, for example Cixutumumab [27], Figitumumab [28], Dalotuzumab [29], Ganitumab [30], R1507 [31], SCH. 717454 [32], AVE1642 [33], BIIB022 [34], LMAb1 [35], and so on. Mechanisms of IGF-IR inhibition by antibodies include blockade of the ligand-receptor complex formation and receptor internalization/degradation [36].

In our study, we characterized the panel of monoclonal antibodies against the ectodomain of the third member of the insulin receptor family, the orphan receptor IRR. Here, we demonstrate that 4D5 antibody binds to the Fn(III)-1 domain of the IRR and therefore represents the first known IRR agonist of the protein nature. Moreover, the effect of this antibody is synergistic with a pH increase. Previously, using multiple chimeras with swapped domains of IR and IRR, we demonstrated that swapping of Fn(III)-1 domain of IRR with the one of IR leads to reduction of pH-sensing activity, but is not critical to the receptor response [16]. Additionally, mutation of the T582 residue of the FnIII-1 domain significantly reduced the IRR activity and was accompanied by a loss of positive cooperativity [15]. This site is located in the region that is structurally equivalent to the insulin binding site 2 of the IR, consisting of C-terminal portion of L2 and the Fn(III)-1 and Fn(III)-2 domains [37,38]. Moreover, previously described IR-activating mAbs 83-14 and 18-44 have epitopes in Fn(III)-1 (a.a. 469–592) and Fn(III)-2/3 domains (a.a. 765–770), respectively [25], which is in good agreement with our results. The results described in this work can be interpreted using the recently published drop-like form of the soluble human IRR ectodomain [18]. Using small angle X-ray scattering (SAXS) method along with atomic force microscopy we have shown that IRR a has drop-like shape at pH 7.4 and 9.0 and there is no significant structural changes during the IRR autophosphorylation, so IRR activation by alkali is probably accompanied by small structural changes inside its drop-like ectodomain, for example rotation. We may speculate that binding of 4D5 antibody to Fn(III)-1 domain can lead to a slight rotation inside the IRR ectodomain resulting in the activation of the receptor, that corresponds the “rotation model” of IR activation [39].

We also demonstrated that antibody 4C2 inhibits the IRR activation by alkali and this antibody binds to the Fn(III)-2/3 domains. Our previous results indicate that the region including residues 646–716 of the FnIII-2 domain of IRR works as “internal ligand” by interacting with adjacent L1 and C domains of the second subunit upon alkali treatment and, together with previously mapped amino residues L135, G188, R244, H318, K319 of L1 and C domains, forms the primary site of pH sensing in IRR [16]. It seems quite similar to the insulin binding site 1 of the IR, consisting of the L1 domain as well as a 12 amino-acid peptide from the insert in Fn(III)-2 domain [37,38].

Interestingly, two different IRR binding assays, immunoprecipitation and immunofluorescence, produced similar, yet slightly different results. Two antibodies 4A2 and inhibitory 4C2 worked well in both assays with wild-type full-length IRR, but showed different profiles in mutant binding. In immunoprecipitation, their binding sites were mapped to the “lethal” mutation in Fn(III)-2/3 domains. Immunocytochemistry data indicated that 4A2 also interacts with L2 domain more distant from the membrane and two-point mutants within it. This difference between “inactive” 4A2 and “inhibitory” 4C2 suggest that the latter blocks IRR activity by restricting juxtamembrane mobility of Fn(III)-2/3 domains possibly by preventing the rotation inside the IRR ectodomain that is mentioned above.

The most important aspect of this study is that the application of domain-specific antibodies to IRR provide yet another line of evidence that the pH sensitivity is an intrinsic property of IRR that has been long questionable. The two structural IRR homologs, IR and IGF-IR, have several cross-reacting proteinaceous ligands with very well studied physiological function and structure. As of now, no natural agonist of IRR has been discovered and until recently, its physiological role was enigmatic. The discovery of IRR alkali sensitivity was especially provocative since other receptor tyrosine kinases typically demonstrate binding of ligands that are peptides or proteins. Thus, a question arises whether the IRR pH sensitivity is an experimental artifact that can be explained by multiple interactions with other proteins or lipids that are in fact protein sensors. Additionally, another hypothetical possibility is that IRR is phosphorylated by another kinase in response to a pH increase. Technically, to assay activation of single and pure molecules of receptor tyrosine kinases is quite challenging. Our approach to use IRR-specific mAbs appeared to be instrumental to establish the direct mechanism of IRR activation. We showed that IRR epitope-specific mAbs can either activate or inhibit IRR autophosphorylation and signaling cascades stimulation. Altogether, our results suggest that the alkali sensing is an intrinsic property of the IRR extracellular region.

## Figures and Tables

**Table 1:** Western blotting of human insulin receptor-related receptor (hIRR), mouse insulin receptor-related receptor (mIRR), and hIRR ectodomain with monoclonal antibodies.

Antibody	With Mercaptoethanol			Without Mercaptoethanol		
	hIRR	mIRR	ectoIRR	hIRR	mIRR	ectoIRR
1D2	−	−	−	−	−	+
4D5	+	−	+	−	−	−
4C2	−	−	−	−	−	+
4A2	−	−	−	−	−	−
3C5	−	−	−	−	−	−
3B4	−	−	−	−	−	+
Anti-IRR ectodomain antibody	+	+	+	+	+	+

**Table 2:** Results of the mapping of the panel of monoclonal antibodies against IRR. The immunoprecipitation data are shown in red, and the immunocytochemistry data are shown in blue.

Protein/Mutant	Relative Activity, %	Аntibody					
		1D2	3C5	4C2	4D5	4A2	3B4
IRR	100	+ +	+ +	+ +	+ +	+ +	− −
L1C	35 [16]	− −	+ +	+ +	+ +	+ +	− −
L2	64 [16]	+ +	+ +	+ +	+ +	+ −	− −
FnIII-1	36 [16]	+ +	− −	+ −	− −	+ −	− −
FnIII-2/3	0 [16]	+ +	− −	− −	+ +	− −	− −
IRR-5A	96 [16]	− −	− −	+ +	+ +	+ +	− −
C-pept-TK(N)	100 [16]	+ +	+ +	+ +	+ +	+ +	− −
MVD	28 [16]	+ +	− −	+ +	+ +	+ −	− −
PV	154 [16]	+ +	+ +	+ +	+ +	+ −	− −
T(A)	64 [15]	+ +	+ +	+ +	+ +	+ +	− −
RL-Fn	100 [15]	+ +	+ +	+ +	+ +	+ −	− −
Result		L1C	Fn(III)-1	Fn(III)-2/3	Fn(III)-1	Fn(III)-2/3	−

## Supplementary Materials

The following are available online at https://www.mdpi.com/2218-273X/10/7/1060/s1, Figure S1: Domain structure of chimeric forms of IRR and mutant forms containing point amino acid substitutions; Figure S2: Results of the Western blotting of control cells (1), hIRR (2), mIRR (3), and purified ectoIRR (4) under reducing conditions using mouse monoclonal antibodies against ectoIRR; Figure S3: Results of the Western blotting of control cells (1), hIRR (2), mIRR (3), and purified ectoIRR (4) under non-reducing conditions using mouse monoclonal antibodies against ectoIRR; Figure S4: Results of ELISA of the antibodies used in this work; Figure S5: Results of the immunoprecipitation of the full-size human IRR and its 10 mutant forms using mouse monoclonal antibodies against ectoIRR; Figure S6: Fluorescence of the HEK293T cells expressing IRR and IRR/IR chimera with L1C domain replacement after the immunocytochemistry using mouse monoclonal antibodies against ectoIRR and secondary anti-mouse Cy3-labeled antibodies; Figure S7: Cross-reactivity test with human IR. Results of the immunoprecipitation of the IRR and IR using mouse monoclonal antibodies against IRR; Figure S8: Cross-reactivity test with human IGF-IR. Results of the immunoprecipitation of the IRR and IGF-IR using mouse monoclonal antibodies against IRR; Figure S9: Cross-reactivity test with mouse IRR; Figure S10: Results of the IRR immunoprecipitation with preliminary incubation of cells in mildly alkaline medium.
