# Supplementary material for: Probing Structure and Function of Alkali Sensor IRR with Monoclonal Antibodies
Source: Biomolecules. 2020 Jul 16;10(7):1060. doi: 10.3390/biom10071060 (PMC7408431; doi:10.3390/biom10071060)
Supplement: Supplementary file 1 [file biomolecules-10-01060-s001.pdf]

Supplementary Materials:

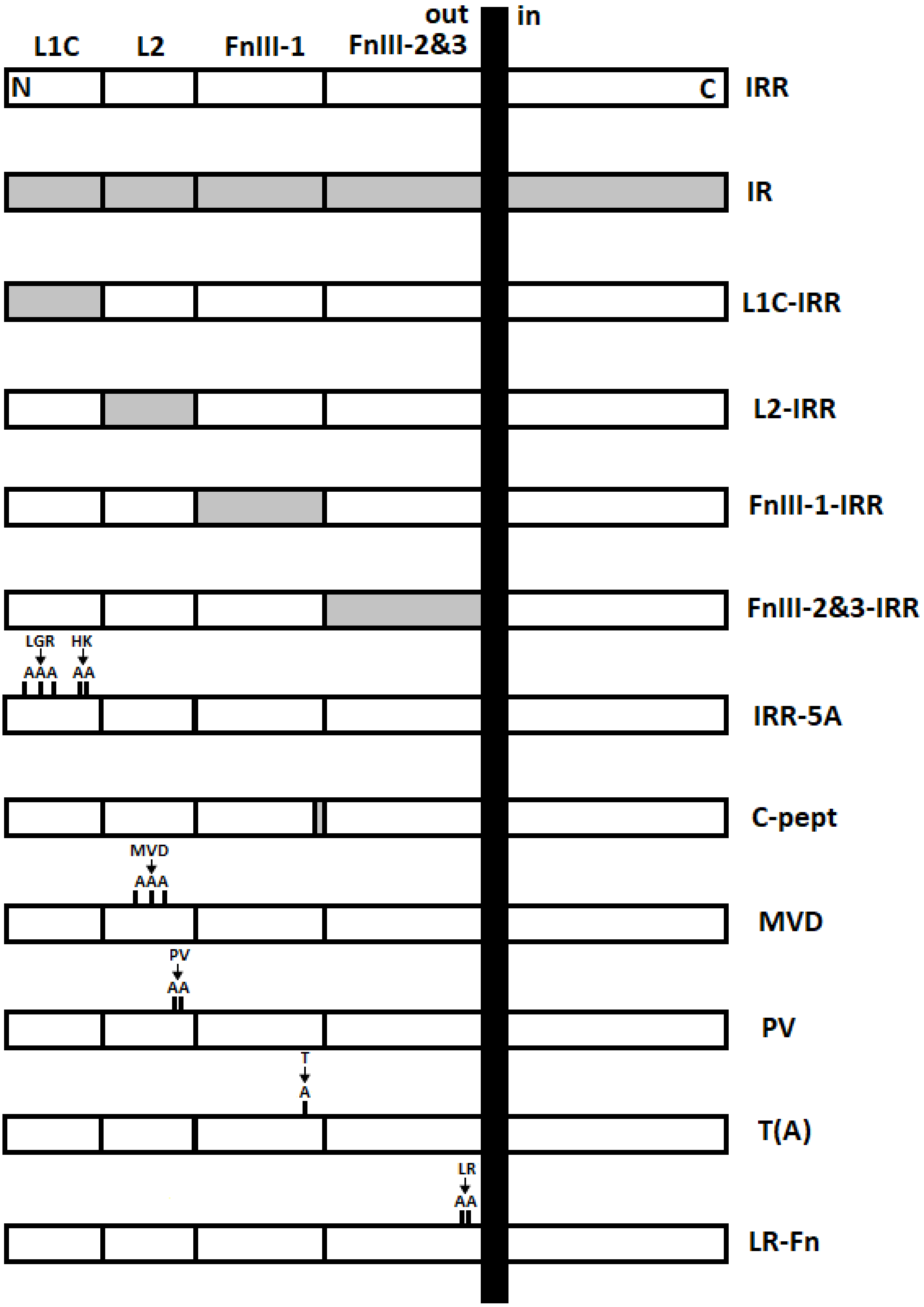

Figure S1. Domain structure of chimeric forms of IRR and mutant forms containing point amino acid substitutions.

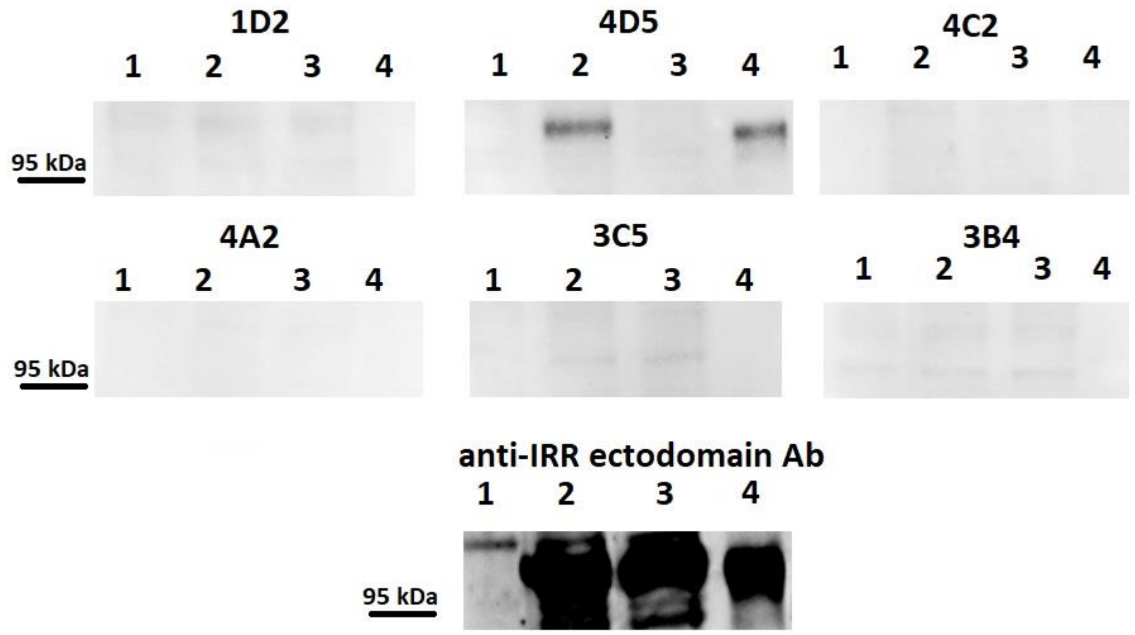

Figure S2. Results of the Western blotting of control cells (1), hIRR (2), mIRR (3) and purified ectoIRR (4) under reducing conditions using mouse monoclonal antibodies against ectoIRR.

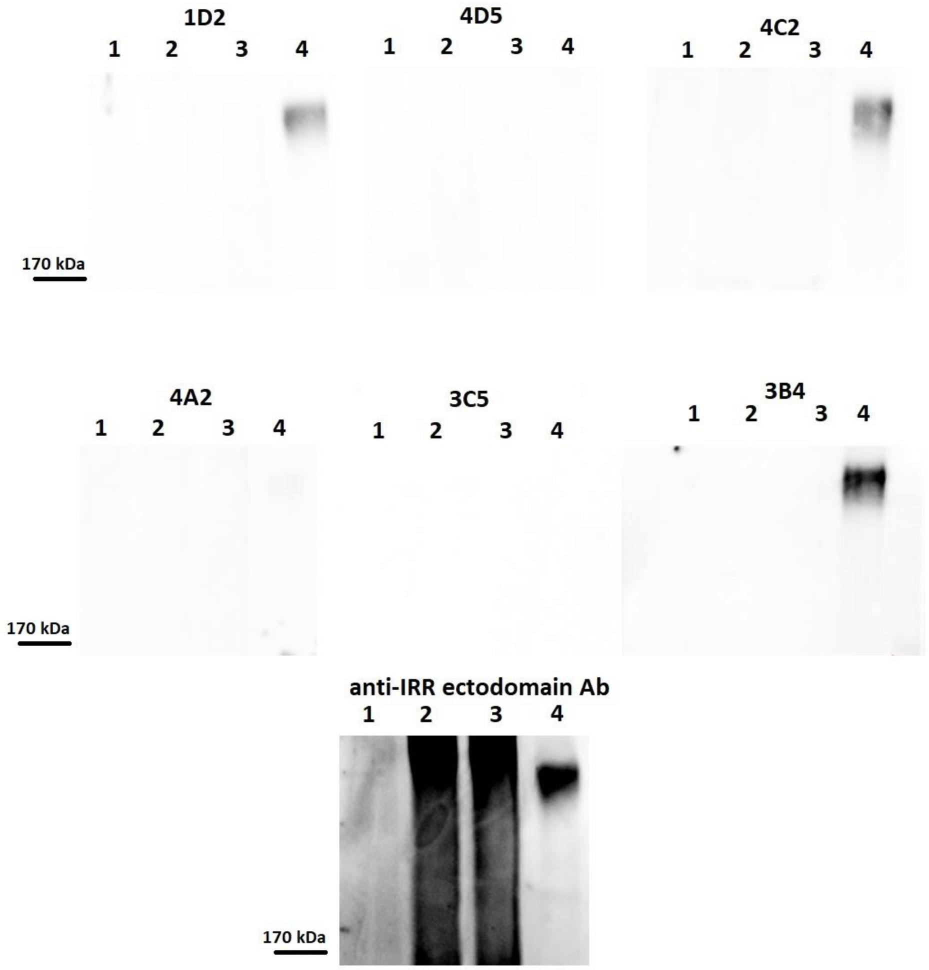

Figure S3. Results of the Western blotting of control cells (1), hIRR (2), mIRR (3) and purified ectoIRR (4) under non-reducing conditions using mouse monoclonal antibodies against ectoIRR.

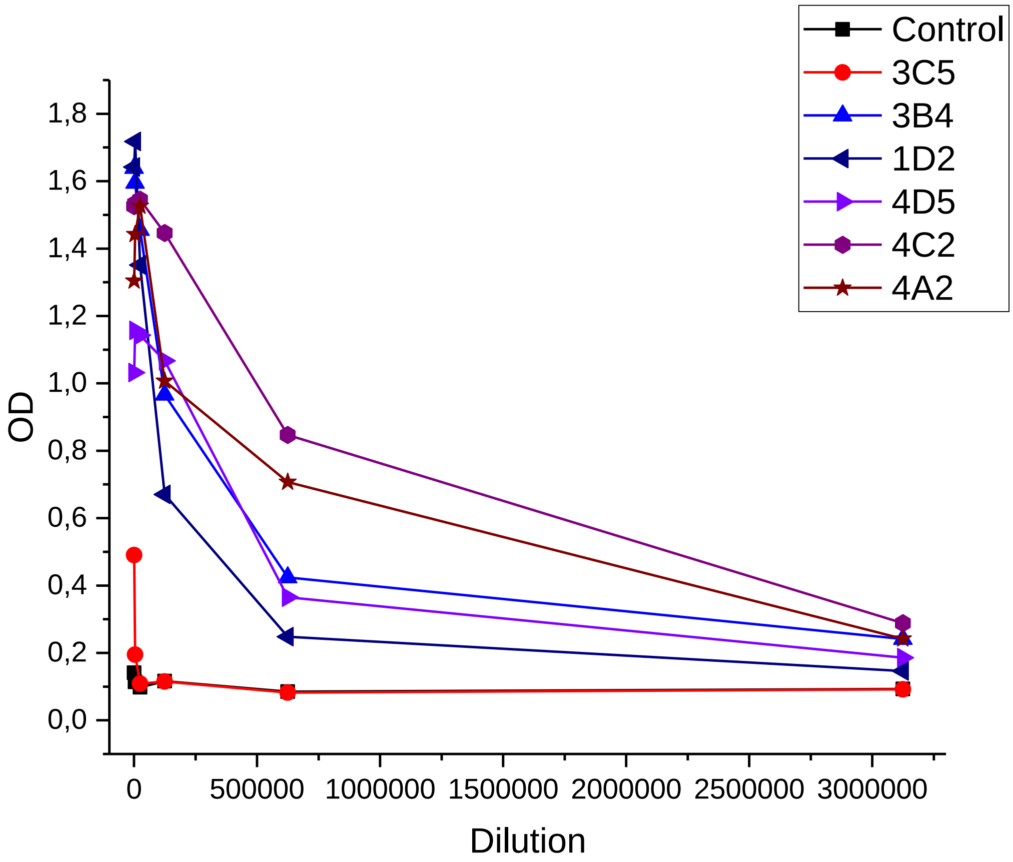

Figure S4. Results of ELISA of the antibodies used in this work. The 96-well plate was treated with 30 μg/mL ectoIRR solution overnight, blocked with 2% BSA solution for 1 hour, then samples of antibodies with different dilution were added to the wells for 2 hours. Wells were washed with PBS and rabbit anti-mouse secondary antibodies conjugated to the alkaline phosphatase were added. After 1 hour of incubation, wells were washed with PBS and PNPP (p-nitrophenyl phosphate) substrate (1 mg/mL) was added. After incubation during 1 hour at RT, ODs of the samples at 405 nm were measured.

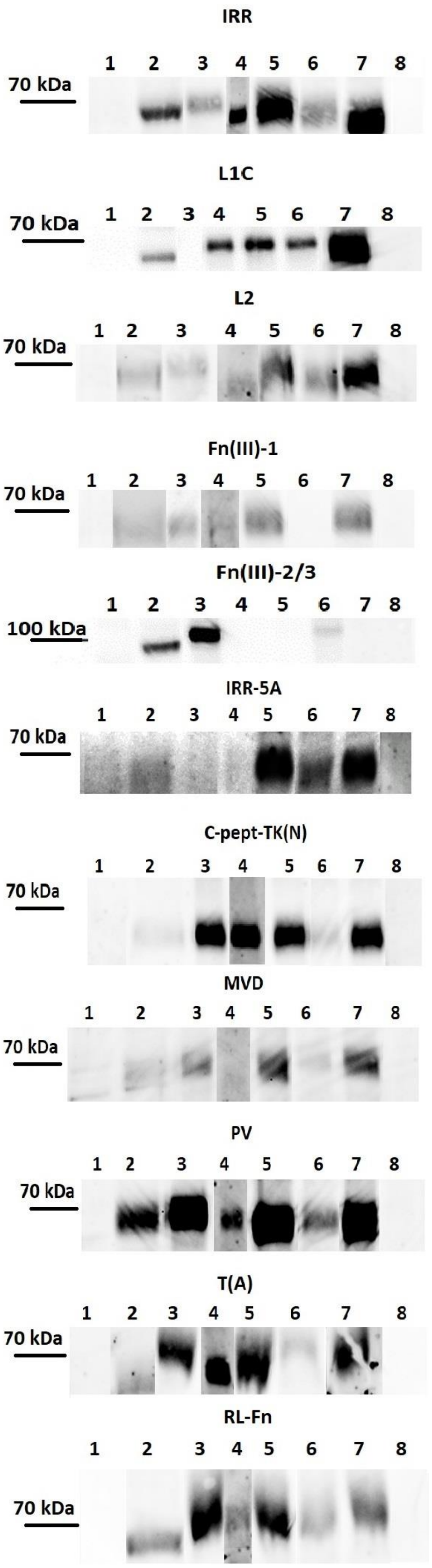

Figure S5. Results of the immunoprecipitation of the full-size human IRR and its 10 mutant forms using mouse monoclonal antibodies against ectoIRR. 1) Control cells; 2) Positive control (lysate of the cells expressing corresponding protein of interest); 3) Precipitation with 1D2 mAbs; 4) Precipitation with 3C5 mAbs; 5) Precipitation with 4C2 mAbs; 6) Precipitation with 4D5 mAbs; 7) Precipitation with 4A2 mAbs; 8) Precipitation with 3B4 mAbs.

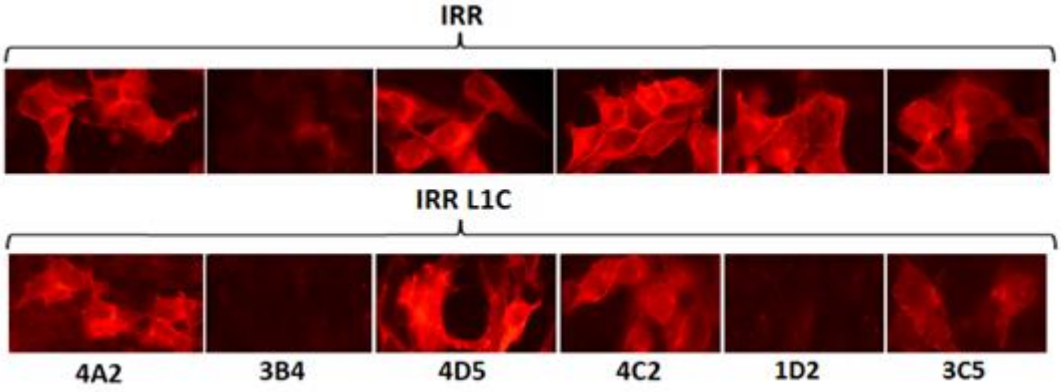

Figure S6. Fluorescence of the HEK293T cells expressing IRR and IRR/IR chimera with L1C domain replacement after the immunocytochemistry using mouse monoclonal antibodies against ectoIRR and secondary anti-mouse Cy3-labeled antibodies.

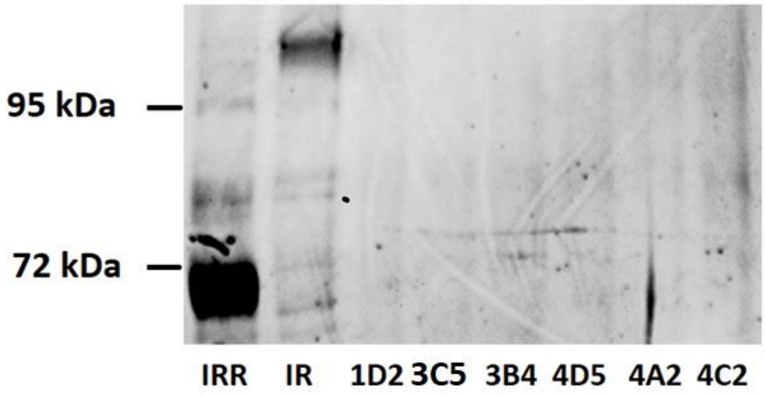

Figure S7. Cross-reactivity test with human IR. Results of the immunoprecipitation of the IRR and IR using mouse monoclonal antibodies against IRR.

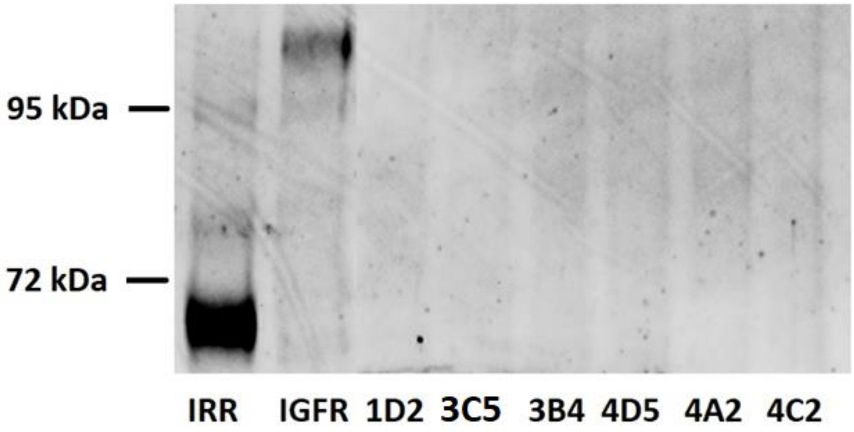

Figure S8. Cross-reactivity test with human IGF-1R. Results of the immunoprecipitation of the IRR and IGF-IR using mouse monoclonal antibodies against IRR.

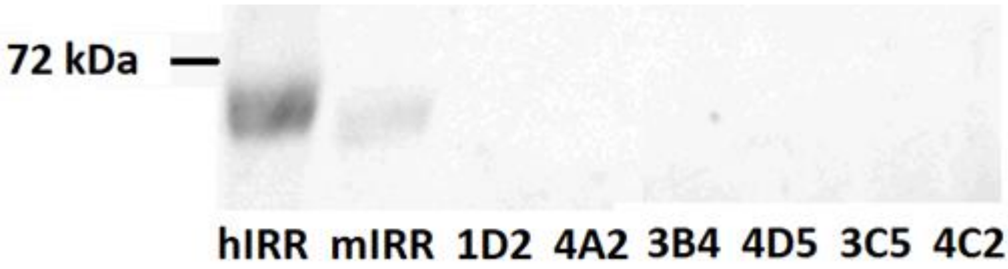

Figure S9. Cross-reactivity test with mouse IRR. Results of the immunoprecipitation of the human IRR and mouse IRR using mouse monoclonal antibodies against human IRR.

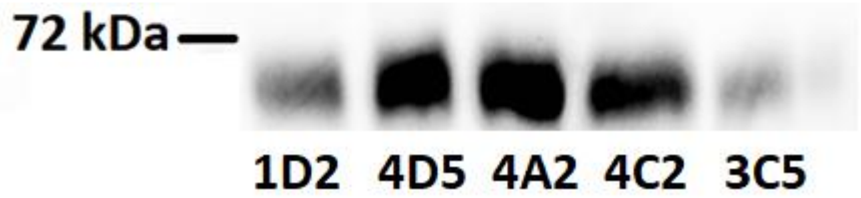

Figure S10. Results of the IRR immunoprecipitation with preliminary incubation of cells in mildly alkaline medium. After the Western blotting, membrane was stained using anti-C-end-IRR antibodies.
